# Associations Between the Modified Food Standard Agency Nutrient Profiling System Dietary Index and Cardiovascular Risk Factors in an Elderly Population

**DOI:** 10.3389/fnut.2022.897089

**Published:** 2022-07-14

**Authors:** Nadine Khoury, Clara Gómez-Donoso, María Ángeles Martínez, Miguel Ángel Martínez-González, Dolores Corella, Montserrat Fitó, J. Alfredo Martínez, Ángel M. Alonso-Gómez, Julia Wärnberg, Jesús Vioque, Dora Romaguera, Ana León-Acuña, Francisco J. Tinahones, José M. Santos-Lozano, Luís Serra-Majem, Paloma Massó Guijarro, Josep A. Tur, Vicente Martín Sánchez, Xavier Pintó, Miguel Delgado-Rodríguez, Pilar Matía-Martín, Josep Vidal, Clotilde Vázquez, Lidia Daimiel, Emili Ros, Maira Bes-Rastrollo, Rocio Barragan, Olga Castañer, Jose D. Torres-Peña, Leyre Notario-Barandiaran, Carlos Muñoz-Bravo, Itziar Abete, Lara Prohens, Naomi Cano-Ibáñez, Lucas Tojal Sierra, José Carlos Fernández-García, Carmen Sayon-Orea, Maria Pascual, Jose V. Sorli, Dolores Zomeño, Patricia J. Peña-Orihuela, Antonio J. Signes-Pastor, F. Javier Basterra-Gortari, Helmut Schröeder, Jordi Salas Salvadó, Nancy Babio

**Affiliations:** ^1^Universitat Rovira i Virgili, Departament de Bioquimica i Biotecnologia, Unitat de Nutrició Humana, Reus, Spain; ^2^Institut d’Investigació Sanitària Pere Virgili (IISPV), Reus, Spain; ^3^Centro de Investigación Biomédica en Red Fisiopatología de la Obesidad y la Nutrición (CIBEROBN), Instituto de Salud Carlos III, Madrid, Spain; ^4^Department of Preventive Medicine and Public Health, IDISNA, University of Navarra, Pamplona, Spain; ^5^Department of Nutrition, Harvard T.H. Chan School of Public Health, Boston, MA, United States; ^6^Department of Preventive Medicine, University of Valencia, Valencia, Spain; ^7^Unit of Cardiovascular Risk and Nutrition, Institut Hospital del Mar de Investigaciones Médicas Municipal d‘Investigació Médica (IMIM), Barcelona, Spain; ^8^Department of Nutrition, Food Sciences, and Physiology, Center for Nutrition Research, University of Navarra, Pamplona, Spain; ^9^Precision Nutrition and Cardiometabolic Health Program, IMDEA Food, CEI UAM + CSIC, Madrid, Spain; ^10^Osakidetza Basque Health Service, Araba University Hospital, Bioaraba Health Research Institute, University of the Basque Country UPV/EHU, Vitoria-Gasteiz, Spain; ^11^Department of Public Health and Psychiatry, Biomedical Research Institute of Malaga (IBIMA), University of Málaga, Málaga, Spain; ^12^CIBER de Epidemiología y Salud Pública (CIBERESP), Instituto de Salud Carlos III, Madrid, Spain; ^13^Instituto de Investigación Sanitaria y Biomédica de Alicante, Universidad Miguel Hernández (ISABIAL-UMH), Alicante, Spain; ^14^Health Research Institute of the Balearic Islands (IdISBa), Palma de Mallorca, Spain; ^15^Department of Internal Medicine, Maimonides Biomedical Research Institute of Cordoba (IMIBIC), Reina Sofia University Hospital, University of Córdoba, Córdoba, Spain; ^16^Department of Endocrinology, Virgen de la Victoria Hospital, Instituto de Investigación Biomédica de Málaga (IBIMA), University of Málaga, Málaga, Spain; ^17^Research Unit, Department of Family Medicine, Distrito Sanitario Atención Primaria Sevilla, Seville, Spain; ^18^Research Institute of Biomedical and Health Sciences (IUIBS), University of Las Palmas de Gran Canaria and Centro Hospitalario Universitario Insular Materno Infantil (CHUIMI), Canarian Health Service, Las Palmas de Gran Canaria, Spain; ^19^Department of Preventive Medicine and Public Health, University of Granada, Granada, Spain; ^20^Preventive Medicine Unit, Universitary Hospital Virgen de las Nieves, Granada, Spain; ^21^Instituto de Investigación Biosanitaria de Granada (ibs.GRANADA), Granada, Spain; ^22^Research Group on Community Nutrition and Oxidative Stress, University of Balearic Islands-IUNICS, Palma de Mallorca, Spain; ^23^Institute of Biomedicine (IBIOMED), University of León, León, Spain; ^24^Lipids and Vascular Risk Unit, Internal Medicine, Hospital Universitario de Bellvitge, Hospitalet de Llobregat, Barcelona, Spain; ^25^Division of Preventive Medicine, Faculty of Medicine, University of Jaén, Jaén, Spain; ^26^Department of Endocrinology and Nutrition, Instituto de Investigación Sanitaria Hospital Clínico San Carlos (IdISSC), Madrid, Spain; ^27^CIBER Diabetes y Enfermedades Metabólicas (CIBERDEM), Instituto de Salud Carlos III (ISCIII), Madrid, Spain; ^28^Department of Endocrinology, Institut d‘Investigacions Biomédiques August Pi Sunyer (IDIBAPS), Hospital Clinic, University of Barcelona, Barcelona, Spain; ^29^Department of Endocrinology and Nutrition, Hospital Fundación Jimenez Díaz, Instituto de Investigaciones Biomédicas IISFJD, University of Autonoma, Madrid, Spain; ^30^Nutritional Control of the Epigenome Group, Precision Nutrition and Obesity Program, IMDEA Food, CEI UAM + CSIC, Madrid, Spain; ^31^Department of Endocrinology and Nutrition, Institut d’Investigacions Biomèdiques August Pi Sunyer (IDIBAPS), Hospital Clínic, Lipid Clinic, Barcelona, Spain; ^32^School of Health Sciences, Blanquerna-Ramon Llull University, Barcelona, Spain; ^33^Department of Endocrinology and Nutrition, Hospital Universitario de Navarra, Pamplona, Spain

**Keywords:** front of pack food labeling, cardiovascular risk factor, body weight, FSAm-NPS dietary index, PREDIMED-Plus study

## Abstract

**Background:**

Helping consumers to improve the nutritional quality of their diet is a key public health action to prevent cardiovascular diseases (CVDs). The modified version of the Food Standard Agency Nutrient Profiling System Dietary Index (FSAm-NPS DI) underpinning the Nutri-Score front-of-pack label has been used in public health strategies to address the deleterious consequences of poor diets. This study aimed to assess the association between the FSAm-NPS DI and some CVD risk factors including body mass index (BMI), waist circumference, plasma glucose levels, triglyceride levels, high-density lipoprotein (HDL) and low-density lipoprotein (LDL) cholesterol, and diastolic and systolic blood pressure.

**Materials and Methods:**

Dietary intake was assessed at baseline and after 1 year of follow-up using a 143-item validated semi-quantitative food-frequency questionnaire. Dietary indices based on FSAm-NPS applied at an individual level were computed to characterize the diet quality of 5,921 participants aged 55–75 years with overweight/obesity and metabolic syndrome from the PREDIMED-plus cohort. Associations between the FSAm-NPS DI and CVD risk factors were assessed using linear regression models.

**Results:**

Compared to participants with a higher nutritional quality of diet (measured by a lower FSAm-NPS DI at baseline or a decrease in FSAm-NPS DI after 1 year), those participants with a lower nutritional quality of diet (higher FSAm-NPS DI or an increase in score) showed a significant increase in the levels of plasma glucose, triglycerides, diastolic blood pressure, BMI, and waist circumference (β coefficient [95% confidence interval]; *P* for trend) (1.67 [0.43, 2.90]; <0.001; 6.27 [2.46, 10.09]; <0.001; 0.56 [0.08, 1.05]; 0.001; 0.51 [0.41, 0.60]; <0.001; 1.19 [0.89, 1.50]; <0.001, respectively). No significant associations in relation to changes in HDL and LDL-cholesterol nor with systolic blood pressure were shown.

**Conclusion:**

This prospective cohort study suggests that the consumption of food items with a higher FSAm-NPS DI is associated with increased levels of several major risk factors for CVD including adiposity, fasting plasma glucose, triglycerides, and diastolic blood pressure. However, results must be cautiously interpreted because no significant prospective associations were identified for critical CVD risk factors, such as HDL and LDL-cholesterol, and systolic blood pressure.

## Introduction

Cardiovascular disease (CVD) is the leading cause of mortality and is considered a major global public health problem (1, 2). According to the estimation in 2019, CVD burden was found to be responsible for 17.9 million deaths worldwide, accounting for approximately 32% of all global deaths, representing a huge economic and social cost (3).

A healthy diet is recognized as a lever for public health by using a modifiable determinant of CVD and other chronic diseases that can be addressed through primary prevention interventions (4). In contrast, an unhealthy diet characterized by an excess of energy, added sugar, salt and saturated fats, and a lack of fruits, vegetables, and fibers has been recognized as an important causal CVD risk factor through the modulation of adiposity and other cardiometabolic risk factors (5).

Over the past decade, different strategies have been proposed to increase adherence to a healthy diet and reduce the risk of CVD. One of the recent initiatives in this regard is the adoption of front-of-pack (FOP) nutrition labels (6). Although labels on the back of the pack are already mandatory in all European countries according to RE 1169/2011 (7), there is evidence that this information is not easily understandable by consumers. In contrast, nutrition labels found on the front of pack of products are considered more helpful and efficient for consumers since nutritional information is summarized and available at a glance (8, 9). FOP labeling aims to help consumers make healthier choices at the point of purchase and to incentivize food manufacturers to reduce the content of nutrients that might compromise diet quality (e.g., salt, saturated fatty acids, and sugar) and/or increase the content of beneficial nutrients (e.g., fibers and vitamins) (10, 11).

Front-of-pack nutrition labels reflect the nutritional quality of food using a nutrient profiling system (NPS) (12). Nutrient profiling is widely used to support public health initiatives to promote healthy eating (12). Among the available nutrient profiling systems, the NPS developed by the UK Food Standard Agency (FSA-NPS) has been consistently validated in Europe (13). This system was originally developed to discriminate foods based on their nutritional composition in the context of television commercials targeting children (14). In France, after some modifications of the FSA-NPS by the French High Council for Public Health (Haut Conseil de la Santé Publique, HCSP) and demonstrating its applicability within the French context, the modified FSA-NPS (FSAm-NPS) was established and a dietary index (DI) based on the FSAm-NPS (FSAm-NPS DI) was developed to validate the algorithm underlying the Nutri-Score FOP label (15, 16). The score for a given food or beverage is calculated by allocating points for the content per 100 g or 100 ml. This algorithm considers both positive scoring components (protein, fiber, percentage of fruit, vegetables, legumes, nuts, rapeseed oil, walnut oil, and olive oil) and negative components (energy, sugars, saturated fatty acids, and salt) (17), and has been proved effective to rank products by nutritional quality and improve food purchases in real-life grocery shopping settings (18).

In addition, several large European prospective studies have shown associations between a higher FSAm-NPS (lower nutritional quality) and an increased risk of cancer (19), CVD (20), cancer mortality (21), CVD mortality (22), and all-cause mortality (23). In contrast, a lower FSAm-NPS (better nutritional quality) has been associated with a lower risk of long-term weight gain and metabolic syndrome incidence in the SU.VI.MAX French cohort study (24, 25). However, there is less information available about the association between the FSAm-NPS DI and CVD risk factors. The aim of this study was to evaluate the association between the FSAm-NPS DI and different CVD risk factors including body mass index (BMI), waist circumference, glucose, triglycerides, HDL- and LDL-cholesterol levels, as well as diastolic and systolic blood pressure in elderly adults with overweight/obesity and metabolic syndrome.

## Materials and Methods

### Study Population and Design: The PREDIMED Plus Study

**FIGURE 1:**
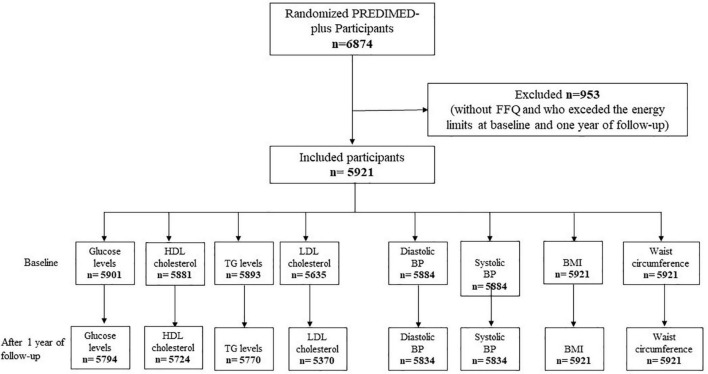
Flow chart of participant’s inclusion.

A prospective analysis was conducted using the data of the PREDIMED-Plus (PREvención con DIeta MEDiterránea) cohort. The PREDIMED-Plus trial is an ongoing randomized, controlled trial conducted in 23 Spanish centers that aims to compare the effect of an intensive weight loss intervention (based on an energy-reduced Mediterranean diet, physical activity promotion, and behavioral support) on the incidence of CVD events, to a control group that receives usual care advice and non-caloric reduced Mediterranean diet recommendations. A detailed description of the PREDIMED-Plus study is available at https://www.predimedplus.com. Between October 2013 and December 2016, 6,874 participants were recruited in Spain and were randomly assigned to either an intensive lifestyle intervention or standard medical care in a 1:1 ratio. Eligible participants were overweight or obese (BMI 27–40 kg/m^2^) men and women aged 55–75 years who met at least three metabolic syndrome criteria as follows: waist circumference > 102 cm in men and >88 cm in women; serum triglycerides ≥ 150 mg/dl or drug treatment for elevated triglycerides; HDL-c < 40 mg/dl in men and <50 mg/dl in women or drug treatment for low HDL-cholesterol; blood pressure ≥ 130/85 mmHg or antihypertensive drug treatment; and fasting plasma glucose level ≥ 100 mg/dl or hypoglycemic treatment and without documented history of CVD. Extensive descriptions of inclusion and exclusion criteria can be found elsewhere (26). All subjects gave written informed consent, and the final protocol and methods were approved by the institutional review boards of each participating center. A total of 953 participants who did not complete a food frequency questionnaire (FFQ) at baseline and after 1 year of follow-up or with total calorie intake outside the pre-specified energy limits (women < 500 and >3,500 kcal/day, and men < 800 and >4,000 kcal/day) were excluded from the analyses (27) Figure 1.

### Dietary Intake Assessment

Dietary intake was assessed at baseline and after 1 year of follow-up using a validated FFQ administered face-to-face by trained dietitians (27). The participants reported their average consumption in terms of frequency and quantity for 143 food and beverage items during the previous year. The frequency of consumption was shown through nine categories ranging from never or almost never to more than six times a day, and a commonly used portion size was specified (e.g., slices, glass, and teaspoons) to indicate serving sizes for each of the items.

The nutrient intake was calculated as the sum of the frequency of daily consumption of each item multiplied by the nutrient composition of the specified serving size according to the Spanish food composition database (28). The total energy intake was also estimated from the quantity and frequency of food and beverage consumption.

### Dietary Index Based on the French Context, the Modified FSA-NPS Computation

The FSAm-NPS is a modified version of the original FSA-NPS (29). Modifications made by the French National Nutrition and Health Program and the French High Council for Public Health impacted the score standards for cheese, added fats, and beverages. For all foods and beverages in the PREDIMED-plus FFQ, the FSAm-NPS was computed per 100 g of the product as follows: 0–40 points was given for the content of critical nutrients that should be limited (0–10 points for each: sugars, saturated fats, sodium, and energy), and 0–15 points was given for the content of beneficial nutrients that should be encouraged (0–5 points for each: fibers, proteins, and the percentage of fruits, vegetables, legumes, nuts, rapeseeds, walnuts, and olive oil that make up the total product). The total score was calculated by subtracting the “negative” (nutrients to avoid) scores from the “positive” (nutrients to promote) scores. As a result, the final FSAm-NPS for each food/beverage was calculated using a discrete continuous scale with a theoretical range of −15 (healthiest) to 40 (least healthy). To generate a dietary index at the individual level, the FSAm-NPS DI was calculated for each participant accounting for their whole diet through energy-weighted means of the FSAm-NPS of all foods and beverages consumed with the following equation:


(1) $$\mathrm{Dietary}\,\mathrm{Index}=\frac{\sum_{i=1}^{n}F\,S\,i\,E\,i}{\sum_{i=1}^{n}E\,i}$$

*where “i” signifies a food or beverage consumed by the participant, “FSi” the food (or beverage) score, “Ei”, the mean daily energy intake from this food (or beverage), and “n” the number of different foods.*

Therefore, a higher FSAm-NPS-DI reflects lower nutritional quality of the individual’s overall diet.

### Outcome (Cardiovascular Diseases Risk Factors) Assessment at Baseline and After 1 Year of Follow-Up

Qualified PREDIMED-Plus staff followed the study protocol to measure anthropometric variables and blood pressure. CVD risk factors such as glucose levels, triglyceride levels, HDL-cholesterol, LDL-cholesterol, diastolic and systolic blood pressure, BMI, and waist circumference were assessed at baseline and after 1 year of follow-up.

#### Waist Circumference

It was measured midway between the lowest rib and the iliac crest using an anthropometric tape, and body weight was measured twice using high-quality electronic calibrated scales and height was measured twice using a wall-mounted stadiometer.

#### Body Mass Index

It was computed by dividing the weight in kilograms by the square of height in meters.

#### Systolic and Diastolic Blood Pressure

They were measured three times using a validated semiautomatic oscillometer (Omron HEM-705CP, Kyoto, Japan) and the mean of repeated measures was used.

#### Fasting Plasma Levels of Glucose, Total Cholesterol, High-Density Lipoprotein-Cholesterol, and Triglycerides

They were measured using standard enzymatic methods. LDL-cholesterol was calculated using the Friedewald formula (whenever triglycerides were less than 300 mg/dl and whenever triglycerides were more than 300 mg/dl, LDL-cholesterol was calculated with the standard method).

### Assessment of Covariates

Covariates were assessed by trained staff in a face-to face interview using self-reported general questionnaires that collect information on socio-demographics (sex, age, marital status, and level of education), lifestyle (smoking habits and physical activity), and medication use. Leisure time physical activity was estimated using the validated Minnesota-REGICOR Short Physical Activity questionnaire (30).

### Statistical Analyses

For our analyses, we used the PREDIMED-Plus database updated in December 2020. Analysis of descriptive baseline characteristics of sociodemographic, dietary, and biomedical variables was carried out and reported as means ± SD or median [P25–P75] and percentages (number) for continuous and categorical variables, respectively.

Participants were categorized by tertiles of the FSAm-NPS DI, ranging from tertile 1 (T1) for the best nutritional quality to tertile 3 (T3) for the lowest nutritional quality. The Chi-square test for categorical variables and one-way ANOVA for continuous variables were used to compare the baseline characteristics between the tertiles.

Linear regression models were fitted to assess the associations [β-coefficient (95% confidence interval (CI)] between FSAm-NPS DI (in tertiles and as continuous variables) and CVD risk factors at baseline. We also used linear regression models to explore the prospective associations between changes in the FSAm-NPS DI (in tertiles and as continuous variable) and changes in CVD risk factors after 1 year of follow-up. Changes in the FSAm-NPS-DI were calculated by subtracting 1 year from the baseline values. Missing data after 1 year of follow-up for BMI (four participants) and diastolic BP (one participant) were imputed using the mean value method.

Three models were fitted as follows: crude model, model 1, and the fully adjusted model. Model 1 was adjusted for age (years), sex, BMI (kg/m^2^), educational level (primary or lower, secondary or academic, or graduate), smoking habit (never, former, or current), total energy consumption (kcal/day), physical activity (METs. min/week), and marital status (married, widowed, single or divorced or separated, or religious). The fully adjusted model was further adjusted for medication for the treatment of hypercholesterolemia, hypertension and diabetes, size of the recruitment centers (<250, 250 to <300, 300 to <400, ≥400), and intervention group. In the prospective analysis, each CVD risk factor was adjusted for its baseline level (in model 1 and fully adjusted).

To indirectly assess the effect of olive oil on the associations with cardiovascular risk factors, we conducted a sensitivity analysis where olive oil was valued −8 (the healthiest value in the score, such as for fruits, vegetables, or legumes) in the computation of the FSAm-NPS-DI.

The statistical significance threshold for the results was set at *p* < 0.05. All analyses were conducted with robust estimates of the variance to correct for intra-cluster correlation and using the Stata 14 software program (StataCorp).

## Results

A total of 5,921 participants (52% men and 48% women, with the mean age of 65 years) were included in this study. Baseline characteristics of the participants overall and according to the tertiles of the FSAm-NPS are shown in Table 1. Participants with a higher FSAm-NPS DI were more likely to be men of younger age, to have a higher level of education, to smoke, to be less physically active, and to have a higher alcohol intake. As expected, participants with a lower FSAm-NPS DI, reflecting a higher nutritional quality (Tertile 1), consumed higher amounts of vegetables, fruits, legumes, unrefined cereals, dairy products, white meat, fish and shellfish, nuts, and fiber. In contrast, participants with a higher FSAm-NPS DI (Tertile 2 and 3), reflecting a lower nutritional quality, consumed higher amounts of refined cereals, biscuits, red meat and processed meat, olive oil, alcohol, and sugar, and showed higher intakes of total energy and saturated fat. In relation to biochemical parameters, the participants in the higher tertile of the FSAm-NPS DI showed higher serum concentrations of triglycerides and LDL-cholesterol and lower levels of HDL-cholesterol (Table 1).

**TABLE 1:** Baseline characteristics of participants overall and by tertiles of FSAm-NPS DI score, PREDIMED-plus study.

		Tertiles of the FSAm-NPS DI			*P*-value
	All *n* = 5,921	T1 Higher quality	T2 Moderate quality	T3 Lower quality	
		*n* = 1,974	*n* = 1,974	*n* = 1,973	
FSAm-NPS DI range	1.51–16.6	1.51–7.37	7.38–8.87	8.88–16.6	<0.001
**Sex**					
Men	51.7 (3,065)	44.0 (869)	51.3 (1,012)	60.0 (1,184)	<0.001
Women	48.3 (2,856)	56 (1,105)	48.7 (962)	40.0 (789)	
Age (years)	65.0 ± 4.89	65.6 ± 4.78	65 ± 4.87	64.5 ± 4.95	<0.001
**Marital status**					
Married	76.8 (4,545)	74.7 (1,475)	77 (1,518)	78.7 (1,552)	0.005
Widowed	10.4 (616)	12.5 (245)	10.4 (206)	8.36 (165)	
Single/divorced/separated/Religious	12.5 (741)	12.6 (249)	12.3 (242)	12.7 (250)	
**Educational level**					
Up to primary	49.7 (2,942)	54.1 (1,066)	50 (986)	45.1 (890)	<0.001
Secondary	28.7 (1,701)	27 (531)	28.2 (557)	31.1 (613)	
University	21.6 (1,278)	19.1 (377)	21.8 (431)	23.9 (470)	
**Smoking status**					
Never	44.7 (2,646)	50.7 (1,000)	44.4 (877)	40 (769)	<0.001
Current	12.4 (734)	9.02 (178)	13.2 (260)	15.0 (296)	
Former	42.9 (2,541)	40.3 (796)	42.4 (837)	46.0 (908)	
Physical activity (MET min/week)	2,508 ± 2,322	2,648 ± 2,408	2,469 ± 2,207	2,411 ± 2,342	0.045
**Waist circumference (cm)**					
Men	111 ± 8.72	110 ± 8.6	111 ± 8.4	111 ± 9.0	0.201
Women	104 ± 9.2	103 ± 9.2	104 ± 9.1	104 ± 9.1	0.12
BMI (kg/m^2^)	32.4 ± 3.44	32.4 ± 3.43	32.5 ± 3.41	32.5 ± 3.46	0.376
**Dietary assessment**					
Vegetables (g/day)	329 ± 137	372 ± 147	331 ± 129	283 ± 120	<0.001
Fruits (g/day)	359 ± 205	419 ± 226	361 ± 194	298 ± 171	<0.001
Legumes (g/day)	20.6 ± 11.0	22.8 ± 12.3	20.5 ± 10.9	18.3 ± 9.21	<0.001
Total cereals (g/day)	151 ± 78	148 ± 79	155 ± 79	149 ± 76	0.006
Refined cereals (g/day)	110 ± 87.8	104 ± 91.0	112 ± 88.7	112 ± 83.3	0.001
Unrefined cereals (g/day)	41.1 ± 63.4	43.88 ± 60.97	42.7 ± 66.3	36.6 ± 62.6	0.001
Biscuits (g/day)	26.8 ± 29.7	13.3 ± 15.1	23.6 ± 21	43.5 ± 38.8	<0.001
Dairy products (g/day)	344 ± 200	385 ± 217	334 ± 189	314 ± 187	<0.001
Red meat and derivatives (g/day)	82.3 ± 44.8	76.93 ± 43.57	83.1 ± 43.46	86.9 ± 46.8	<0.001
White meat intake (g/day)	62 ± 34	67.7 ± 36.3	62.6 ± 33.5	55.4 ± 31.2	<0.001
Fish and shellfish intake (g/day)	102 ± 47.4	113 ± 50.6	102 ± 44.8	91.4 ± 43.9	<0.001
Nuts intake (g/day)	15 ± 17	21.2 ± 21.6	14 ± 14.4	9.99 ± 11.4	<0.001
Total olive oil (g/day)	40.1 ± 16.8	33.4 ± 15.2	42.3 ± 15.6	44.7 ± 17.2	<0.001
Refined olive oil (g/day)	8.15 ± 15.1	6.22 ± 12.1	7.96 ± 15.1	10.3 ± 17.4	<0.001
Virgin olive oil (g/day)	31.9 ± 20.7	27.2 ± 18.2	34.3 ± 20.6	34.4 ± 22.2	<0.001
Alcohol (g/day)	11.2 ± 15.1	8.92 ± 13.42	10.9 ± 14.3	13.7 ± 17	<0.001
Dietary fiber intake (g/day)	26.1 ± 8.72	28.7 ± 8.94	26.2 ± 8.53	23.5 ± 7.88	<0.001
Ultra-processed food (g/day)	158 ± 158	107 ± 117	148 ± 137	218 ± 186	<0.001
Total sugar intake (g/day)	6.73 ± 12	4.32 ± 8.76	6.67 ± 11.7	9.21 ± 14.3	<0.001
Total energy (kcal/day)	2,367 ± 550	2,207 ± 519	2,363 ± 521	2,531 ± 560	<0.001
Saturated fat intake (g/day)	26.2 ± 8.45	22.2 ± 6.83	26.0 ± 7.30	30.5 ± 8.93	<0.001
Sodium intake (mg/day)	2,426 ± 773	2,340 ± 770	2,427 ± 756	2,509 ± 784	<0.001
**Biomedical parameters**					
Glucose levels, mg/dL (*n* ± 5,901)	113 ± 29	114 ± 30.2	113 ± 28.7	113 ± 27.9	0.28
TG levels, mg/dL (*n* ± 5,893)	135 [102–178]	136 [102–177]	133 [100–179]	137 [106–178]	0.011
HDL-cholesterol, mg/dL (*n* ± 5,881)	47 [40–55]	48 [41–56]	47 [41–55]	45 [39–53]	<0.001
LDL-cholesterol, mg/dL (*n* ± 5,635)	117 [96–140]	115 [94–140]	118 [97–140]	118 [98–141]	0.028
Systolic BP, mmHg (*n* ± 5,884)	139 ± 16.9	139 ± 16.9	140 ± 17.24	140 ± 16.45	0.119
Diastolic BP, mmHg (*n* ± 5,884)	80.7 ± 9.88	80.4 ± 9.49	80.8 ± 10.14	82 ± 10	0.209

*FSAm-NPS, Food Standards Agency Nutrient Profiling System (modified version) Dietary Index; BMI, body mass index; TG, triglycerides; HDL, high-density lipoprotein; LDL, low-density lipoprotein; BP, blood pressure. Data were expressed as means ± SD or median [P25–P75] and percentages (number) for continuous and categorical variables, respectively. P-values for comparisons were tested by the one-way ANOVA or chi-square test, as appropriate according to the FSAm-NPS tertiles.*

The cross-sectional association between the FSAm-NPS DI and CVD risk factors at baseline is shown in Table 2. Compared to those participants with a lower FSAm-NPS DI (T1) reflecting a healthier diet, those with a higher FSAm-NPS DI (T3) showed lower HDL-cholesterol levels (β: −0.77 [95% CI = −1.47, −0.06]; *P* for trend = 0.027). This association was consistent for the HDL-cholesterol and FSAm-NPS DI as a continuous variable (β: −0.21 [95% CI = −0.37, −0.05]). A direct association was observed between the FSAm-NPS DI as continuous and baseline BMI and waist circumference (β: 0.08 [95% CI = 0.03, 0.13] and β: 0.22 [95% CI = 0.09, 0.35], respectively).

**TABLE 2:** Association between FSAm-NPS DI and CVD risk factors at baseline, and β coefficient (95% CI).

	Tertiles of the FSAm-NPS DI			R-squared	*P*-trend	FSAm-NPS DI at baseline (continuous)
	T1	T2	T3			
**Glucose levels (mg/dL)**						
*n*	1,967	1,967	1,967	0.04	0.177	5,901
Crude Model	*Ref*	−1.31 (−3.14, 0.53)	−1.25 (−3.07, 0.57)	1.84	0.059	−0.21 (−0.63, 0.20)
Model 1	*Ref*	−1.57 (−3.40, 0.27)	−1.80 (−3.68, 0.07)	30.5	0.832	−0.36 (−0.79, 0.07)
Fully adjusted	*Ref*	−0.24 (−1.80, 1.32)	0.16 (−1.41, 1.73)			0.11 (−0.25, 0.47)
**HDL-cholesterol (mg/dL)**						
*n*	1,961	1,960	1,960	0.5	<0.001	5,897
Crude Model	*Ref*	−0.52 (−1.25, 0.22)	−1.98 (−2.73, −1.24)	15.4	0.128	−0.49 (−0.66, −0.33)
Model 1	*Ref*	0.19 (−0.49, 0.88)	−0.52 (−1.24, 0.19)	0.17	0.027	−0.15 (−0.31, 0.01)
Fully adjusted	*Ref*	−0.01 (−0.69, 0.67)	−0.77 (−1.47, −0.06)			−0.21 (−0.37, −0.05)
**TG levels (mg/dL)**						
*n*	1,965	1,964	1,964	0.15	0.004	5,893
Crude Model	*Ref*	1.89 (−2.77, 6.55)	7.15 (2.35, 11.95)	2.88	0.387	1.46 (0.44, 2.49)
Model 1	*Ref*	−0.94 (−5.62, 3.74)	1.96 (−2.97, 6.89)	3.35	0.238	0.19 (−0.89, 1.26)
Fully adjusted	*Ref*	−0.36 (−5.04, 4.31)	2.75 (−2.18, 7.68)			0.36 (−0.72, 1.44)
**LDL-cholesterol (mg/dL)**						
*n*	1,879	1,878	1,878	0.12	0.027	5,635
Crude Model	*Ref*	2.53 (0.45, 4.61)	2.33 (0.26, 4.39)	4.04	<0.001	0.44 (−0.01, 0.89)
Model	*Ref*	3.12 (1.06, 5.17)	3.66 (1.57, 5.75)	26	0.052	0.75 (0.29, 1.22)
Fully adjusted	*Ref*	1.59 (−0.23, 3.41)	1.76 (−0.05, 3.57)			0.35 (−0.05, 0.75)
**Diastolic BP (mmHg)**						
*n*	1,962	1,961	1,961	0.05	0.076	5,884
Crude Model	*Ref*	0.39 (−0.23, 1.01)	0.55 (−0.06, 1.16)	6.52	0.18	0.16 (0.02, 0.30)
Model 1	*Ref*	−0.06 (−0.67, 0.55)	−0.41 (−1.02, 0.20)	7.58	0.063	−0.08 (−0.22, 0.06)
Fully adjusted	*Ref*	−0.14 (−0.75, 0.47)	−0.57 (−1.18, 0.04)			−0.11 (−0.25, 0.03)
**Systolic BP (mmHg)**						
*n*	1,962	1,961	1,961	0.07	0.059	5,884
Crude Model	*Ref*	0.87 (−0.20, 1.95)	1.01 (−0.04, 2.06)	3.36	0.159	0.18 (−0.05, 0.42)
Model 1	*Ref*	0.83 (−0.24, 1.90)	0.79 (−0.28, 1.87)	5.29	0.201	0.12 (−0.13, 0.36)
Fully adjusted	*Ref*	0.50 (−0.56, 1.56)	0.72 (−0.35, 1.78)			0.10 (−0.15, 0.34)
**BMI (kg/m^2^)**						
*n*	1,974	1,974	1,973	0.03	0.17	5,921
Crude Model	*Ref*	0.10 (−0.12, 0.31)	0.15 (−0.07, 0.37)	3.18	0.063	0.06 (0.01, 0.11)
Model 1	*Ref*	0.12 (−0.09, 0.33)	0.22 (−0.01, 0.44)	4.27	0.054	0.08 (0.03, 0.13)
Fully adjusted	*Ref*	0.13 (−0.08, 0.34)	0.22 (0.00, 0.44)			0.08 (0.03, 0.13)
**Waist circumference (cm)**						
*n*	1,979	1,979	1,979	0.06	<0.001	5,921
Crude Model	*Ref*	0.70 (0.10, 1.29)	1.87 (1.27, 2.47)	65.4	0.803	0.51 (0.38, 0.65)
Model 1	*Ref*	−0.21 (−0.57, 0.15)	0.04 (−0.32, 0.41)	17	0.381	0.03 (−0.05, 0.11)
Fully adjusted	*Ref*	0.12 (−0.44, 0.67)	0.60 (0.03, 1.18)			0.22 (0.09, 0.35)

*FSAm-NPS, Food Standards Agency Nutrient Profiling System (modified version) Dietary Index; CVD, cardiovascular disease; CI, confidence interval; HDL, high-density lipoprotein; TG, triglycerides; LDL, low-density lipoprotein; BP, blood pressure; BMI, body mass index. Linear regression models were fitted. Model 1: adjusted for age, sex, physical activity, smoking status, BMI, total energy intake at baseline, education level, and marital status. Fully adjusted: Model 1 additionally adjusted for medication for treatment of hypercholesterolemia, of hypertension and of diabetes and size of the recruitment centers (<250, 250 to <300, 300 to <400, ≥400). All analyses were conducted with robust estimates of the variance to correct for intra-cluster correlation. R-squared was multiplied by 100.*

Associations between 1 year changes in the FSAm-NPS DI and changes in the CVD risk factors are shown in Table 3. Compared to participants with a score change resulting in a lower FSAm-NPS DI (T1), those participants with a higher FSAm-NPS DI (T3) after 1 year of follow-up showed a significant increase in the levels of plasma glucose, triglycerides, diastolic blood pressure, BMI, and waist circumference (β: 1.67 [95% CI = 0.43, 2.90]; *P* for trend < 0.001; β: 6.27 [95% CI = 2.46, 10.09]; *P* for trend < 0.001; β: 0.56 [95% CI = 0.08, 1.05]; *P* for trend = 0.001; β: 0.51 [95% CI = 0.41, 0.60]; *P* for trend < 0.001; β: 1.19 [CI = 0.89, 1.50]; *P* for trend < 0.001, respectively).

**TABLE 3:** Association between 1-year change of FSAm-NPS and changes of the CVD risk factors levels after 1 year of follow-up, and β coefficient (95% CI).

	Tertiles of one-year FSAm-NPS DI change			R-squared	*P*-trend	FSAm-NPS DI one-year change (continuous)
	T1	T2	T3			
**Glucose levels change (mg/dL)**						
*n*	1,932	1,931	1,931	0.27	<0.001	5,794
Crude Model	Ref	2.51 (1.17, 3.86)	2.47 (1.06, 3.88)	18.4	<0.001	0.50 (0.20, 0.81)
Model 1	Ref	2.06 (0.82, 3.31)	2.56 (1.29, 3.83)	23.3	<0.001	0.42 (0.14, 0.70)
Fully adjusted	Ref	1.76 (0.54, 2.98)	1.67 (0.43, 2.90)			0.35 (0.09, 0.62)
**HDL-cholesterol change (mg/dL)**						
*n*	1,908	1,908	1,908	0.1	0.021	5,724
Crude Model	Ref	−0.40 (−0.87, 0.07)	−0.54 (−1.00, −0.08)	8.44	0.019	−0.09 (−0.19, 0.0.1)
Model 1	Ref	−0.28 (−0.73, 0.17)	−0.53 (−0.98, 0.09)	9.31	0.024	−0.03 (−0.12, 0.07)
Fully adjusted	Ref	−0.16 (−0.60, 0.29)	−0.27 (−0.72, 0.18)			−0.02 (−0.12, 0.07)
**TG levels change (mg/dL)**						
*n*	1,924	1,923	1,923	0.43	<0.001	5,770
Crude Model	Ref	4.43 (0.04, 8.81)	11.0 (6.70, 15.25)	25.3	<0.001	2.75 (1.87, 3.63)
Model 1	Ref	3.85 (0.04, 7.65)	8.84 (5.06, 12.63)	26.5	<0.001	1.81 (1.05, 2.56)
Fully adjusted	Ref	2.75 (−1.06, 6.56)	6.27 (2.46, 10.09)			1.75 (1.00, 2.51)
**LDL-cholesterol change (mg/dL)**						
*n*	1,790	1,790	1,790	0.03	0.673	5,370
Crude Model	Ref	−0.77 (−2.59, 1.05)	0.41 (−1.35, 2.17)	19.2	0.282	−0.04 (−0.42, 0.33)
Model 1	Ref	−0.92 (−2.56, 0.72)	−0.86 (−2.46, 0.74)	21.6	0.287	−0.34 (−0.69, 0.00)
Fully adjusted	Ref	−1.06 (−2.68, 0.56)	−0.88 (−2.48, 0.72)			−0.34 (−0.68, 0.00)
**Diastolic BP change (mmHg)**						
*n*	1,945	1,945	1,944	0.13	0.007	5,834
Crude Model	Ref	0.50 (−0.02, 1.03)	0.72 (0.19, 1.24)	20	0.002	0.17 (0.05, 0.28)
Model 1	Ref	0.58 (0.12, 1.05)	0.76 (0.28, 1.24)	20.8	0.001	0.14 (0.04, 0.24)
Fully adjusted	Ref	0.45 (−0.02, 0.92)	0.56 (0.08, 1.05)			0.15 (0.04, 0.25)
**Systolic BP change (mmHg)**						
*n*	1,945	1,945	1,944	0.09	0.03	5,834
Crude Model	Ref	0.45 (−0.49, 1.39)	1.08 (0.11, 2.05)	22.8	0.028	0.31 (0.10, 0.51)
Model 1	Ref	0.70 (−0.13, 1.53)	0.96 (0.10, 1.82)	23.6	0.041	0.18 (0.00, 0.37)
Fully adjusted	Ref	0.45 (−0.39, 1.28)	0.49 (−0.39, 1.36)			0.17 (−0.01, 0.35)
**BMI change (kg/m^2^)**						
*n*	1,974	1,974	1,973	4.04	<0.001	5,921
Crude Model	Ref	0.41 (0.31, 0.51)	0.77 (0.67, 0.87)	5.08	<0.001	0.17 (0.14, 0.19)
Model 1	Ref	0.40 (0.30, 0.50)	0.75 (0.65, 0.85)	16.1	<0.001	0.11 (0.09, 0.13)
Fully adjusted	Ref	0.27 (0.18, 0.36)	0.51 (0.41, 0.60)			0.11 (0.09, 0.13)
**Waist circumference change (cm)**						
*n*	1,967	1,966	1,966	2.77	<0.001	5,899
Crude Model	Ref	1.22 (0.90, 1.55)	2.09 (1.76, 2.41)	9.74	<0.001	0.47 (0.40, 0.54)
Model 1	Ref	0.11 (0.79, 1.43)	1.99 (1.68, 2.31)	15.5	<0.001	0.29 (0.23, 0.36)
Fully adjusted	Ref	0.73 (0.42, 1.03)	1.19 (0.89, 1.50)			0.28 (0.22, 0.35)

*FSAm-NPS, Food Standards Agency Nutrient Profiling System (modified version) Dietary Index; CVD, cardiovascular disease; CI, confidence interval; HDL, high-density lipoprotein; TG, triglycerides; LDL, low-density lipoprotein; BP, blood pressure; BMI, body mass index. Linear regression models were fitted. Model 1: adjusted for age, sex, physical activity, smoking status, BMI, total energy intake at baseline, education level and marital status and each CVD risk factor was adjusted to its level at baseline. Fully adjusted: Model 1 additionally adjusted for medication for treatment of hypercholesterolemia, of hypertension and of diabetes, size of the recruitment centers (<250, 250 to <300, 300 to <400, ≥400) and the intervention groups. All analyses were conducted with robust estimates of the variance to correct for intra-cluster correlation. R-squared was multiplied by 100.*

**FIGURE 2:**
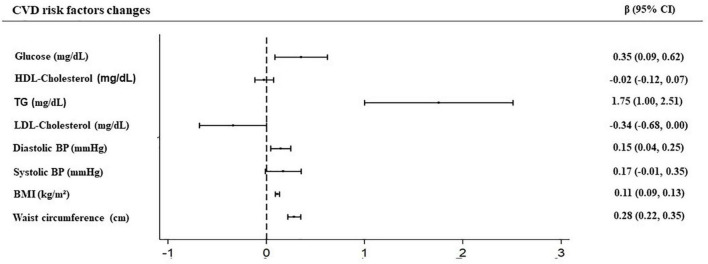
Prospective associations between dietary index based on the French context, the modified FSA-NPS (FSAm-NPS DI) and cardiovascular diseases (CVD) risk factors changes after 1 year of follow-up.

In Figure 2, we present the β coefficient (95% CI) for the prospective associations between changes in the FSAm/NPS DI (continuous) and changes in the CVD risk factors after 1 year of follow-up. Consistent with the results described above, positive associations were found between changes in the FSAm-NPS DI and changes in the glucose and triglyceride levels, diastolic blood pressure, BMI, and waist circumference after 1 year of follow-up. Non-significant association was observed in the case of HDL-cholesterol, LDL-cholesterol, and systolic blood pressure changes. Nevertheless, after 1 year of follow-up, 79.36 and 19.4% of the population remained in the recommended levels of HDL and LDL-cholesterol, 12.82 and 8.9% of the participants showed increased levels, and only 7.82 and 10.9% of the participants showed a decrease in HDL-cholesterol or LDL-cholesterol, respectively (data not shown).

The cross-sectional and longitudinal associations remained significant when olive oil was considered the healthiest food option in the computation of the FSAm-NPS DI (data not shown).

## Discussion

To the best of our knowledge, this study is one of the first to evaluate the prospective associations between the nutritional quality of diet assessed using the FSAm-NPS DI (algorithm underpinning the Nutri-Score FOP label) and different CVD risk factors. Findings of this study showed that the consumption of foods with higher scores of FSAm NPS (foods with less favorable rating in the Nutri-Score scale) was associated with unfavorable changes in CVD risk factors after 1 year of follow-up, specifically with an increase in adiposity (BMI and waist circumference) and the levels of plasma glucose, triglycerides, and diastolic blood pressure.

Our results are partially in line with the SU.VI.MAX study where the authors assessed the prospective association between the FSAm-NPS DI and metabolic syndrome and its components (24). Even though, these authors found significant and positive association between the FSAm-NPS DI and the incidence of metabolic syndrome, when the components were assessed individually, only systolic and diastolic blood pressure showed a significant association. In our study, we also found significant and positive association with diastolic blood pressure, but not for systolic. These results may be explained because the FSAm-NPS DI considers the salt content of food that has been proved to be related to hypertension (31). However, we cannot discard that other factors of the score, as well as their synergistic effect, may also explain these results on blood pressure. In contrast to the SU.VI.MAX study, we have demonstrated significant prospective associations with triglycerides and fasting glucose. These results in relation to triglycerides and glucose may be explained because the participants in our study were elderly Mediterranean individuals (aged between 55 and 75 years) with overweight/obesity and metabolic syndrome. Therefore, as they were already at the risk of CVD, the results of our study could be influenced by their pre-existing risk and conditions. In contrast, in the SU.VI.MAX study, the participants were younger and healthier (women aged between 35 and 60 years and men aged between 45 and 60 years).

In our study, no prospective associations were observed between changes in the FSAm-NPS DI and changes in HDL-cholesterol or LDL-cholesterol concentrations. Even though, in the cross-sectional analysis, we found an association between HDL-cholesterol and FSAm-NPS DI at the baseline, the lack of association in the prospective analysis might be explained because most of the population did not show changes in the levels of HDL-cholesterol after 1 year of follow-up. In a previous study, analysis conducted in the SU.VI.MAX cohort reported a negative association between the FSAm-NPS DI and LDL-cholesterol concentration (16). According to the authors, the absence of association or the negative association observed in the case of the LDL-cholesterol could be partly explained by the fact that, even when saturated fat is considered in the score, the FSAm-NPS DI does not distinguish between fatty acid subtypes (monounsaturated or polyunsaturated fatty acids) (25).

In our study, a higher 1-year increase in the FSAm-NPS, reflecting a decrease in the nutritional quality of diet, was also positively associated with increases in adiposity measured by BMI and waist circumference. These findings are in line with other studies using other nutritional quality indexes (32, 33), and with other two studies analyzing the FSAm-NPS. In the SU.VI.MAX French cohort, a positive association between the FSAm-NPS DI and body weight and BMI gain (24) was found in both men and women. In addition, an increased risk of obesity was observed only in men after 13 years of follow-up. A study using data from the French NutriNet-Santé cohort has recently analyzed the prospective associations between different nutrient profiling systems (the original Food Standards Agency nutrient profiling system and three variants, Food Standards Australia New Zealand Nutrient Profiling Scoring Criterion (NPSC), Health Star Rating NPS and the French NPS (HCSP-NPS), and adiposity markers and overweight/obesity risk. The results showed that participants with a dietary index reflecting lower diet nutritional quality (irrespective of the nutrient profiling used) were more likely to increase the BMI over time and had an increased risk to develop overweight/obesity (34). It is important to remark that whilst differences were small, the French FSAm-NPS one appeared to show a significantly greater association with the risk of overweight compared to other nutrient profile scores. It is important to highlight that the reclassification of olive oil in the FSAm-NPS computation only induced small effects in our study, whereas previous evidence from the SUN cohort found that the reclassification of olive oil strengthened the associations between the FSAm-NPS and total mortality (23).

In the FSAm-NPS, total energy, salt, saturated fat, and sugar are considered negative components whereas fiber, protein, fruits and vegetables, legumes, and some vegetable oils are considered positive. The FSAm-NPS DI resumes the FSAm-NPS scores of all food items mostly consumed by an individual, by assigning points based on the consumption of foods, food groups, or nutrients relevant to the risk of chronic diseases. All these nutrients or food groups have been demonstrated to be related to CVD risk factors in numerous studies (5, 35, 36). Particularly, added sugar and saturated fat have been previously associated with higher levels of fasting blood glucose and triglycerides (37, 38). Therefore, the observed associations are consistent with the nature of the FSAm-NPS and with previous findings regarding diet and CVD.

Furthermore, there is also increasing evidence that ultra-processed foods (UPF) have detrimental effects on CVD risk (39), In this sense, we should bear in mind that the FSAm-NPS DI and the NOVA classification (based on degree of food processing) are two different complementary approaches to assess nutritional quality and healthiness (40). Indeed, in our study, those participants allocated in the highest FSAm-NPS DI tertiles had higher UPF consumption. It is acknowledged that the FSAm-NPS DI does not cover all the health dimensions of food (e.g., food processing, additives, and presence of pesticides). It considers neither the added sugar included in food nor the monounsaturated or polyunsaturated fat because this score has been based on those nutrients that, in Europe, are mandatory to be disclosed in the list of nutrients in the food labeling. However, at the moment, there is no classification system including all these nutrients and dimensions of food in a single indicator. Therefore, FSAm-NPS focuses on the nutritional dimension and serves as the underlying system of some front-of-pack labels to allow consumers to easily compare foods belonging to the same category. Importantly, FSAm-NPS has the advantage of considering a large number of elements from a nutritional point of view, in particular the content, per 100 g of food of fruits and vegetables (proxy of the amount of antioxidants, vitamins, and minerals), legumes, nuts, proteins (e.g., proxy of the amount of calcium and iron), olive oil, rapeseed oil, and walnut oils.

Overall, our results support the suitability of the FSAm-NPS as a nutritional quality indicator aimed at improving diets and preventing the development of chronic diseases. Further prospective studies analyzing the effect of modifying the nutritional quality of diet through changes in this dietary index are warranted in the future to confirm our results.

### Strengths and Limitations

Our study has several limitations that deserve to be discussed. First, the results cannot be generalized to other populations since participants included in the analysis were elderly Mediterranean individuals with overweight/obesity and metabolic syndrome. Therefore, as they were already at the risk of CVD, the results of this study could be influenced by this condition. Second, the assessment of food intake through a FFQ is prone to possible measurement errors. However, despite this limitation, food-based FFQs have been widely used as a tool in epidemiological studies since the 1990s (41). Third, we cannot rule out bias due to unmeasured potential confounders related to the risk of CVD.

This study also has some strengths such as its prospective design, which reduces the possibility of reverse causation bias, the control for several potential confounding factors, the large sample size, and the extensive data collected by trained staff.

In conclusion, the results of this prospective cohort study suggest that the consumption of foods with higher FSAm-NPS (reflecting a lower nutritional quality) is associated with an increase in some CVD risk factors (adiposity, fasting plasma glucose, triglycerides, and diastolic blood pressure). However, no significant associations were identified for critical CVD risk factors such as HDL-cholesterol, LDL-cholesterol, and systolic blood pressure.

## Data Availability Statement

The datasets presented in this article are not readily available because the datasets generated and analyzed during the current study are not publicly available due to data regulations and for ethical reasons, considering that this information might compromise research participants’ consent because our participants only gave their consent for the use of their data by the original team of investigators. However, collaboration for data analyses can be requested by sending a letter to the PREDIMED-Plus Steering Committee (predimed_plus_scommittee@googlegroups.com). The request will then be passed to all the members of the PREDIMED-Plus Steering Committee for deliberation. Requests to access the datasets should be directed to predimed_plus_scommittee@googlegroups.com.

## Ethics Statement

The studies involving human participants were reviewed and approved by JW: CEI Provincial de Málaga-Servicio Andaluz de Salud (O01_feb_PR2), José Lapetra: CEI de los Hospitales Universitarios Virgen Macarena y Virgen del Rocío-Servicio Andaluz de Salud (PI13/00673), JAM: CEIC Universidad de Navarra (053/2013), DR: CEI de las Illes Balears – Conselleria de Salut Direcció General de Salut Publica i Consum (IB 2242/14 PI), MF: CEIC Parc de Salut Mar y IDIAP Jordi Gol (PI13/120), JSS: CEIC del Hospital Universitari Sant Joan de Reus y IDIAB Jordi Gol (13-07-25/7proj2), Aurora Bueno: CEI de la Provincia de Granada- Servicio Andaluz de Salud (MAB/BGP/pg), CV: CEIC de la Fundacion Jiménez Díaz (EC 26-14/IIS-FJD), MM-G: CEIC Universidad de Navarra (053/2013), Fernando Aros: CEIC Euskadi (PI2014044), DC: CEIC Corporativo de Atención Primaria de la Comunitat Valenciana (2011-005398-22), LS-M: CEI Humana de la Universidad de las Palmas de Gran Canaria (CEIH-2013-07), XP: CEIC del Hospital de Bellvitge (PR240/13), José López Miranda: CEI de Cordoba-Junta de Salud (3078), José María Ordovás: CEI de la Fundación IMDEA Alimentación (PI-012), PM-M: CEIC Hospital Clínico San Carlos de Madrid-Piloto-CEIC Servicio Madrileño de salud-General (30/15), FT: CEI Provincial de Málaga-Servicio Andaluz de Salud, JT: CEI de las Illes Balears – Conselleria de Salut Direcció General de Salut Publica i Consum (IB 2251/14 PI), JoV: CEIC del Hospital Clínic de Barcelona (HCB/2017/0351), JeV: CEIC del Hospital General Universitario de Alicante (CEIC PI2017/02), M-DR: CEIC de la Investigación Biomédica de Andalucía (CCEIBA), VM: CEI de la Universidad de León (TICA-ULE-014-2015), and Ramon Estruch: CEIC del Hospital Clínic de Barcelona (HCB/2016/0287). The patients/participants provided their written informed consent to participate in this study.

## Author Contributions

NK, CG-D, MM, JSS, and NB conceived and designed the study. MM, DC, MF, JAM, ÁA-G, JW, JV, DR, FT, JS-L, LS-M, JT, VM, XP, MD-R, PM-M, JVi, CV, LD, and ER conducted data acquisition. NK, CG-D, and NB performed statistical analyses. NK, CG-D, MM, JSS, and NB carried out interpretation of the data for the study. All authors were involved in draft redaction, revision for important intellectual content, read, and approved the final manuscript.

## Conflict of Interest

JSS served on the board of the International Nut and Dried Fruit Council and receives grant support through this institution. He also served in the Executive Committee of the Instituto Danone, Spain, and on the Scientific Committee of the Danone International Institute. He received research support from the Patrimonio Comunal Olivarero, Spain, and Borges S.A., Spain. He received consulting fees or travel expenses from Eroski Foundation, the Instituto Danone, Spain, Mundipharma and Abbot Laboratories. ER reports grants, personal fees, non-financial support, and others from California Walnut Commission and Alexion, personal fees, non-financial support, and others from Ferrer International and Danone, and personal fees from Amarin, other than the submitted study. The remaining authors declare that the research was conducted in the absence of any commercial or financial relationships that could be construed as a potential conflict of interest.

## Publisher’s Note

All claims expressed in this article are solely those of the authors and do not necessarily represent those of their affiliated organizations, or those of the publisher, the editors and the reviewers. Any product that may be evaluated in this article, or claim that may be made by its manufacturer, is not guaranteed or endorsed by the publisher.
